# Effects of TECAR Therapy on Body Surface Temperature and Longissimus Dorsi Muscle Tone in Thoroughbred Racehorses: A Pilot Study

**DOI:** 10.3390/ani16152300

**Published:** 2026-07-24

**Authors:** Weronika Grzegorczyk, Maria Soroko-Dubrovina, Maciej Dobrowolski, Dobrosława Grabska, Krzysztof D. Dudek

**Affiliations:** 1 Independent Researcher, 31-586 Kraków, Poland; wer.grzegorczyk@gmail.com; 2 Institute of Animal Breeding, Wrocław University of Environmental and Life Sciences, Chelmońskiego 38C, 51-160 Wrocław, Poland; maciej.dobrowolski@upwr.edu.pl (M.D.); 125920@student.upwr.edu.pl (D.G.); 3 Clinical Research Support Center, 4th Military Clinical Hospital in Wrocław, 50-981 Wrocław, Poland; krzysztof.dudek@pwr.edu.pl

**Keywords:** capacitive and resistive electric transfer, horse, thermographic examination, muscle tension, longissimus dorsi, physiotherapy

## Abstract

Capacitive and resistive electric transfer (TECAR) therapy is increasingly used in equine physiotherapy; however, objective evidence regarding its immediate effects on equine back muscles remains limited. This pilot study aimed to evaluate the effects of TECAR therapy on body surface temperature and muscle tone of the longissimus dorsi muscle in Thoroughbred racehorses. A randomized, single-blinded experimental study was conducted in 20 clinically healthy Thoroughbred racehorses aged 2–3 years. Horses were randomly assigned to either a TECAR treatment group (*n* = 10) or a sham-treatment control group (*n* = 10). Thermographic imaging and palpation-based muscle tone assessment were performed before treatment, immediately after treatment, and 10 min post-treatment in the thoracolumbar region (Th15–L6). Differences between groups and changes over time were analyzed using repeated-measures ANOVA for temperature data and Friedman’s test with post hoc Wilcoxon signed-rank tests for palpation scores. TECAR therapy was associated with a significant increase in body surface temperature, with mean values rising from 25.3 ± 1.5 °C before treatment to 28.9 ± 1.3 °C immediately after treatment (*p* < 0.001) and remaining elevated after 10 min (27.6 ± 1.3 °C; *p* = 0.002). In contrast, only minor temperature changes were observed in the sham group. Palpation assessment demonstrated a significant reduction in muscle tone following TECAR therapy, with median scores decreasing from 3 [2–3] before treatment to 0 [0–1] immediately afterward (*p* < 0.001). These findings indicate that TECAR therapy induces an immediate thermal response, which persists for at least 10 min, and is associated with reduced longissimus dorsi muscle tone in racing horses. Further studies are warranted to investigate its clinical applications and long-term effects in equine rehabilitation.

## Simple Summary

Back muscle tension is a common problem in racehorses and may negatively affect comfort, movement quality, and athletic performance. Capacitive and resistive electric transfer (TECAR) therapy is a physiotherapeutic method that uses radiofrequency energy to generate heat within tissues and is increasingly applied in equine rehabilitation. However, scientific evidence describing its immediate effects in horses remains limited. In this study, we evaluated how a single TECAR treatment affected body surface temperature and muscle tension in healthy Thoroughbred racehorses. Twenty horses were assigned either to an active TECAR treatment group or to a control group receiving a sham procedure. Horses receiving TECAR therapy showed a clear increase in body surface temperature immediately after treatment, and this effect remained visible 10 min later. At the same time, muscle tension assessed by palpation was markedly reduced. Only minor temperature changes and no meaningful changes in muscle tension were observed in the control horses. These findings indicate that TECAR therapy increases body surface temperature over the treated thoracolumbar region and reduces palpation-assessed back muscle tone in Thoroughbred racehorses. The results provide new information about the immediate effects of TECAR therapy and support further research into its use in equine rehabilitation and sports medicine.

## 1. Introduction

Capacitive and resistive electric transfer (TECAR) therapy, also known as capacitive–resistive electric transfer (CRET), is a non-invasive radiofrequency diathermy modality that has gained increasing attention in both human and veterinary rehabilitation medicine [1,2,3]. The technique employs high-frequency electromagnetic currents, typically ranging from 300 kHz to 1 MHz, delivered between an active and a return electrode to induce endogenous heat generation within biological tissues [4,5,6]. As the applied energy encounters tissue-specific electrical impedance, it is converted into thermal energy, resulting in controlled deep tissue heating without the need for external heat sources [7,8]. TECAR therapy can be delivered in two distinct modes—capacitive (CAP) and resistive (RES)—which differ in their interaction with tissue composition and electrical properties. The capacitive mode primarily targets superficial, low-resistance tissues with high water and electrolyte content, such as skeletal muscles and vascular structures. In contrast, the resistive mode preferentially affects deeper, high-resistance tissues, including tendons, fascia, cartilage, and bone. This differential energy distribution enables selective modulation of both superficial and deep tissues, allowing treatment to be tailored according to therapeutic objectives and tissue characteristics [9]. Beyond its thermal effects, radiofrequency stimulation has been reported to influence cellular metabolism and biological processes associated with tissue repair, regeneration, and recovery [10]. Through the combined action of deep tissue heating and potential bio-stimulatory effects, TECAR therapy has emerged as a promising therapeutic modality in the management of musculoskeletal disorders, particularly in the rehabilitation of soft tissue injuries [2,3]. Although no major adverse events have been reported following TECAR therapy in the available literature, evidence regarding its long-term safety and potential adverse effects, particularly in veterinary medicine, remains limited [6].

The physiological and clinical effects of TECAR therapy have been increasingly explored in the field of human rehabilitation, with a growing body of evidence supporting its therapeutic potential across a range of musculoskeletal conditions [11,12]. Clinical studies have demonstrated beneficial outcomes in patients with chronic low back pain [13,14], chronic neck pain [15], knee osteoarthritis [16], and shoulder impingement syndrome [17]. Reported effects include reductions in pain intensity, improvements in functional performance, and enhanced quality of life. In addition, emerging evidence indicates that TECAR therapy may facilitate recovery from sports-related musculoskeletal disorders by alleviating pain and promoting functional restoration [3]. Recent investigations have also suggested a role for TECAR therapy in accelerating post-exercise neuromuscular recovery following strenuous eccentric exercise, highlighting its potential application in sports medicine and athletic rehabilitation [18].

The growing adoption of TECAR therapy in human rehabilitation has stimulated interest in its application within veterinary physiotherapy. However, despite increasing interest, the clinical evidence supporting TECAR therapy in veterinary medicine remains considerably more limited than that available in human rehabilitation. In recent years, several experimental and clinical studies have examined the effects of TECAR therapy in horses [19,20,21,22]. Most of these studies investigated locomotor performance or clinical outcomes [19,20,21], whereas only the recent study by Calle-González et al. [22] focused on the thermal response of the equine thoracolumbar region using infrared thermography.

Available evidence suggests that TECAR therapy can induce measurable biomechanical and physiological adaptations in equine athletes. In horses affected by thoracolumbar pain, CRET therapy has been associated with pain reduction and improved spinal flexibility, as evidenced by increases in dorsoventral and total accelerometric power following treatment [19]. Similarly, the application of CRET therapy prior to exercise has been shown to modify locomotor parameters in Standardbred trotters, including increases in speed, stride length, and overall locomotor activity, indicating potential benefits for muscle performance and neuromuscular function [20]. Moreover, treadmill-based studies have demonstrated alterations in gait characteristics following CRET treatment in exercised horses [21]. Recent thermographic investigations further revealed that moderate- and high-intensity CRET protocols produce significant increases in body surface temperature within the thoracolumbar region, confirming the capacity of this modality to elicit measurable thermal responses in equine tissues [22].

Despite these promising findings, current evidence remains limited regarding the functional consequences of TECAR-induced thermal effects on skeletal muscle. Most equine studies have focused primarily on thermographic or biomechanical outcomes, while the influence of TECAR therapy on the mechanical properties of treated muscles has received comparatively little attention. Consequently, the relationship between treatment-induced temperature elevation and clinically relevant changes in muscle tone, stiffness, and muscular function remains poorly understood. Addressing this knowledge gap is of particular importance, as the proposed therapeutic benefits of TECAR therapy extend beyond tissue heating and include facilitation of muscle relaxation, reduction in muscle stiffness, and enhancement of neuromuscular performance [5,18,23]. However, controlled experimental studies investigating the association between TECAR-induced thermal responses and changes in palpation-assessed muscle tone in clinically healthy horses are currently lacking. Therefore, the aim of the present study was to evaluate the effects of TECAR therapy on both body surface temperature and palpation-assessed muscle tone of the longissimus dorsi muscle in clinically healthy Thoroughbred racehorses. We hypothesized that TECAR therapy would induce a significant increase in body surface temperature accompanied by a measurable reduction in muscle tone within the treated thoracolumbar region.

## 2. Materials and Methods

The study protocol was reviewed by the Local Ethics Committee for Animal Experiments in Wrocław, Poland (Resolution No. 013/2026, 15 April 2026), which determined that ethical approval was not required, as TECAR therapy is a routinely applied physiotherapeutic procedure in horses and the study did not constitute an animal experiment under applicable legislation. Owner consent was obtained from Partynice Racecourse (Wrocław, Poland) prior to the enrollment of the horses.

### 2.1. Animals and Data Collection

The present study was designed as a randomized, single-blinded experimental trial. Twenty clinically healthy Thoroughbred racehorses aged 2–3 years (11 mares and 9 stallions) were included. All horses were housed at the Partynice Racecourse (Wrocław, Poland), trained by the same trainer, and maintained under identical management and training conditions. Prior to enrollment, all horses underwent routine clinical and musculoskeletal examinations performed by the same experienced equine veterinarian to confirm the absence of lameness and active orthopedic disorders. Lameness evaluation was based on observation of movement and flexion testing to determine its presence and severity [24]. In addition, the thoracolumbar region was assessed using palpation and mobility tests [25].

The horses were assigned to either the TECAR treatment group (*n* = 10; 6 mares and 4 stallions) or the sham group (*n* = 10; 5 mares and 5 stallions) using stratified block randomization, with sex as the stratifying factor to maintain a balanced distribution of mares and stallions between the two groups. Randomization was performed before the experimental procedures to minimize selection bias and ensure comparable baseline characteristics between groups.

On the examination day, horses in the treatment group underwent active TECAR therapy, whereas horses in the sham group received an identical procedure with the applicator positioned in the same manner but without activation of the device. Thermographic imaging followed by palpation assessment was performed before treatment, immediately after treatment, and 10 min after treatment in both groups to evaluate changes in body surface temperature and muscle tone of the longissimus dorsi muscle, according to the methodology described by Calle-González et al. [22].

### 2.2. TECAR Therapy

TECAR therapy was performed using a TECARIS VET device (Astar, Bielsko-Biała, Poland). The treatment was applied once to each horse in the study group over the left longissimus dorsi muscle within the thoracolumbar region (Th15–L6). The treatment area was identified using palpable anatomical landmarks, including the dorsal midline, the bases of the thoracic dorsal spinous processes adjacent to the ribs, and the transverse processes of the lumbar vertebrae, corresponding to the Th15–L6 region. A standardized treatment area of approximately 25 cm^2^ was used for all horses. The boundaries of the treatment region were marked with tape to ensure repeatability and consistency of application. During the procedure, each horse was treated individually in its own box and restrained by a qualified handler to ensure safety and minimize movement. The hair coat in the treatment area was not clipped prior to treatment and all horses had comparable short hair coats typical of Thoroughbred racehorses in active training. A conductive TECAR gel was applied to the skin surface in a standardized amount (approximately 10 mL) to ensure adequate energy transfer.

All procedures were performed by the same operator according to a standardized treatment protocol with a fixed duration of 25 min per horse, ensuring methodological consistency. The protocol incorporated both capacitive (CAP) and resistive (RES) modes, delivered at a radiofrequency of 500 kHz with power levels applied within the ranges of 8–36% (36–162 VA) for CAP and 16–58% (24–87 W) for RES. The applicator was moved continuously over the predefined treatment area using consistent hand movements and light, uniform pressure to maintain optimal tissue contact and ensure homogeneous energy distribution. In the TECAR sham group, the same procedure was performed, including applicator placement, gel application, treatment duration, and hand movements; however, the device remained switched off.

### 2.3. Thermographic Examination

Thermographic examinations were performed using a calibrated VarioCam HR infrared camera (uncooled microbolometer focal plane array; sensor resolution 640 × 480 pixels; spectral range 7.5–14 μm; noise equivalent temperature difference <20 mK at 30 °C; measurement uncertainty ±1% of the overall temperature range; InfraTec, Dresden, Germany). The examination protocol was based on standardized procedures previously described in equine thermography studies [26,27,28]. All horses were examined at rest prior to their daily training session. To minimize environmental influences, including air drafts and direct sunlight, examinations were conducted in a closed stable under controlled environmental conditions. Ambient temperature was 12 ± 1.5 °C and relative humidity was 66 ± 4%, measured using a TES 1314 thermometer (TES, Taipei, Taiwan). Horses underwent a 20 min acclimatization period before image acquisition. All thermographic examinations and TECAR procedures were performed in the horses’ own boxes under the same controlled environmental conditions. The examination area was cleaned of dirt and debris 10 min before each measurement to minimize potential thermal artifacts.

**Figure 1:**
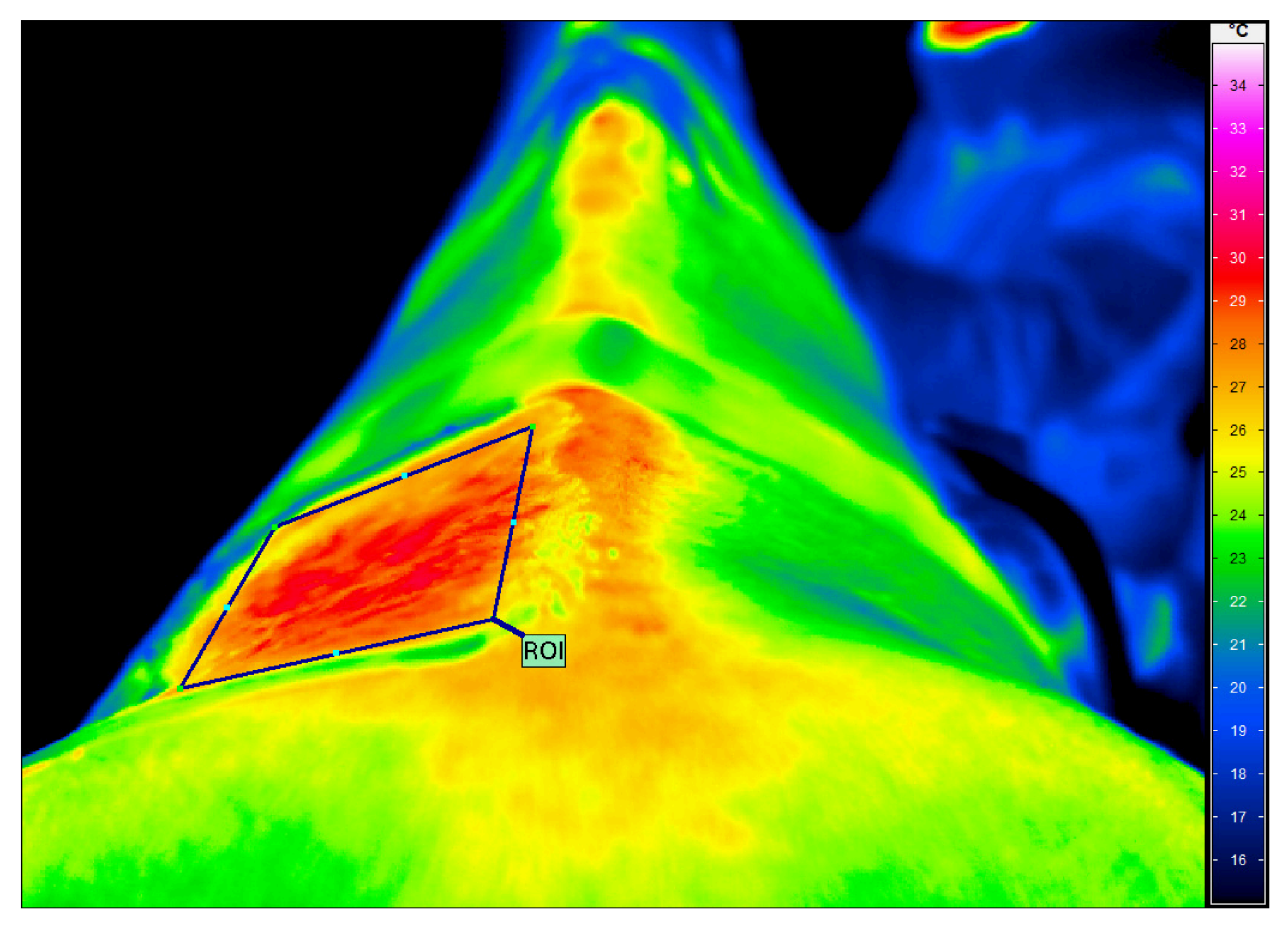
Thermographic image of the thoracolumbar region (Th15–L6) acquired immediately following TECAR therapy of the longissimus dorsi muscle. The outlined rectangular region of interest (ROI) represents the area used for determination of the mean body surface temperature (ROI = 28.27 °C).

All thermographic images of the back were acquired from the dorsal aspect (Figure 1) by the same operator to ensure measurement consistency. The distance between the camera and the horse was maintained constant throughout the study, and emissivity was set at ε = 1 for all measurements. No visible layer of conductive gel remained on the skin surface immediately after treatment or at the 10 min follow-up thermographic assessment.

Thermal analysis was performed using IRBIS 3 Professional software (InfraTec, Dresden, Germany). Mean temperature was determined by a single examiner from a standardized rectangular region of interest (ROI) positioned over the thoracolumbar portion of the longissimus dorsi muscle (Th15–L6). The average body surface temperature (Tavg) obtained from the ROI was used for subsequent analyses.

Mean body surface temperature was assessed at three predefined time points: before treatment (AVG_pre), immediately after treatment (AVG_post), and 10 min after treatment (AVG_10min). These measurements enabled the evaluation of both the immediate and short-term thermal effects of TECAR therapy and were used consistently throughout all analyses.

### 2.4. Palpation Examination

Palpation examination was performed unilaterally over the longissimus dorsi muscle within the thoracolumbar region (Th15–L6), corresponding to the area in which TECAR therapy was applied. The assessment was conducted to evaluate changes in muscle tone and tenderness associated with the intervention. Muscle tone of the longissimus dorsi muscle was evaluated using a palpation scoring system based on the scale described by Varcoe-Cocks et al. [29]: 0 = soft muscle with low tone; 1 = normal muscle tone; 2 = increased muscle stiffness without pain; 3 = stiffness and/or pain accompanied by mild muscle spasms without movement of the horse; 4 = pain associated with muscle spasms and a localized movement response (e.g., pelvic tilt or extension response); and 5 = marked pain with muscle spasms and pronounced behavioral reactions (e.g., ears pinned back or kicking).

All horses were examined and scored by the same experienced veterinarian, who was blinded to group allocation and unaware of whether the horse had received active TECAR therapy or sham treatment. To ensure consistency of the examination, the same standardized palpation technique and pressure were applied throughout the study. Palpation scores were recorded at three predefined time points: before treatment (PAL_pre), immediately after treatment (PAL_post), and 10 min after treatment (PAL_10min).

### 2.5. Statistical Analysis

All statistical analyses were performed using STATISTICA version 13.3 (TIBCO Software Inc., Palo Alto, CA, USA), JASP version 0.19.3 (JASP Team, Amsterdam, The Netherlands), and Microsoft Excel (Microsoft Corporation, Redmond, WA, USA). Statistical analyses were performed according to the type and distribution of the data. Body surface temperature data were expressed as mean ± standard deviation (Mean ± SD), whereas palpation scores were reported as median and interquartile range (Me [IQR]). Prior to repeated-measures ANOVA, the assumptions of normality and homogeneity of variance were assessed. Normality was evaluated using the Shapiro–Wilk test together with visual inspection of Q–Q plots and histograms. Homogeneity of variance was assessed using Levene’s and Bartlett’s tests. When the assumption of equal variances was violated, Welch’s correction was applied. For body surface temperature measurements, Student’s *t*-tests were used to compare the TECAR and sham groups at each time point. Changes in temperature over time (AVG_pre, AVG_post, and AVG_10min) within each group were evaluated using repeated-measures analysis of variance (ANOVA). When repeated-measures ANOVA revealed a significant effect, pairwise comparisons between time points were performed using Tukey’s post hoc test. As palpation scores represented ordinal data, non-parametric methods were applied. Changes in palpation scores over time (PAL_pre, PAL_post, and PAL_10min) within each group were assessed using Friedman’s test. When Friedman’s test revealed a significant overall effect, pairwise comparisons between time points were performed using the Wilcoxon signed-rank test. Effect sizes were additionally calculated to quantify the magnitude of the observed effects. Hedges’ g was reported for between-group comparisons, partial eta-squared (η^2^p) for repeated-measures ANOVA, and Kendall’s W for Friedman’s test. The significance level (α) was set at 0.05 and served as the predefined reference threshold for interpretation of the obtained *p*-values.

## 3. Results

### 3.1. Thermographic Examination

Changes in body surface temperature were observed across the three assessment time points (AVG_pre, AVG_post, and AVG_10min) in both groups. In the TECAR-treated group, body surface temperature increased markedly immediately after treatment and remained elevated 10 min later, despite a slight decline from the post-treatment value. In contrast, the TECAR sham group exhibited only a minor increase in temperature immediately after the procedure, followed by a return toward baseline values at the final assessment.

No significant differences in mean body surface temperature were detected between the study and sham groups before treatment (AVG_pre: 25.3 ± 1.5 °C vs. 25.0 ± 1.3 °C, respectively; *p* = 0.61). Immediately after treatment, the study group demonstrated significantly higher temperatures than the sham group (AVG_post: 28.9 ± 1.3 °C vs. 25.8 ± 1.1 °C; *p* < 0.001). The mean increase in body surface temperature immediately following the procedure was 3.1 °C in the study group, compared with 0.8 °C in the sham group. A significant difference between groups remained evident 10 min after treatment (AVG_10min: 27.6 ± 1.3 °C vs. 25.4 ± 0.9 °C; *p* = 0.002) (Table 1).

**Figure 2:**
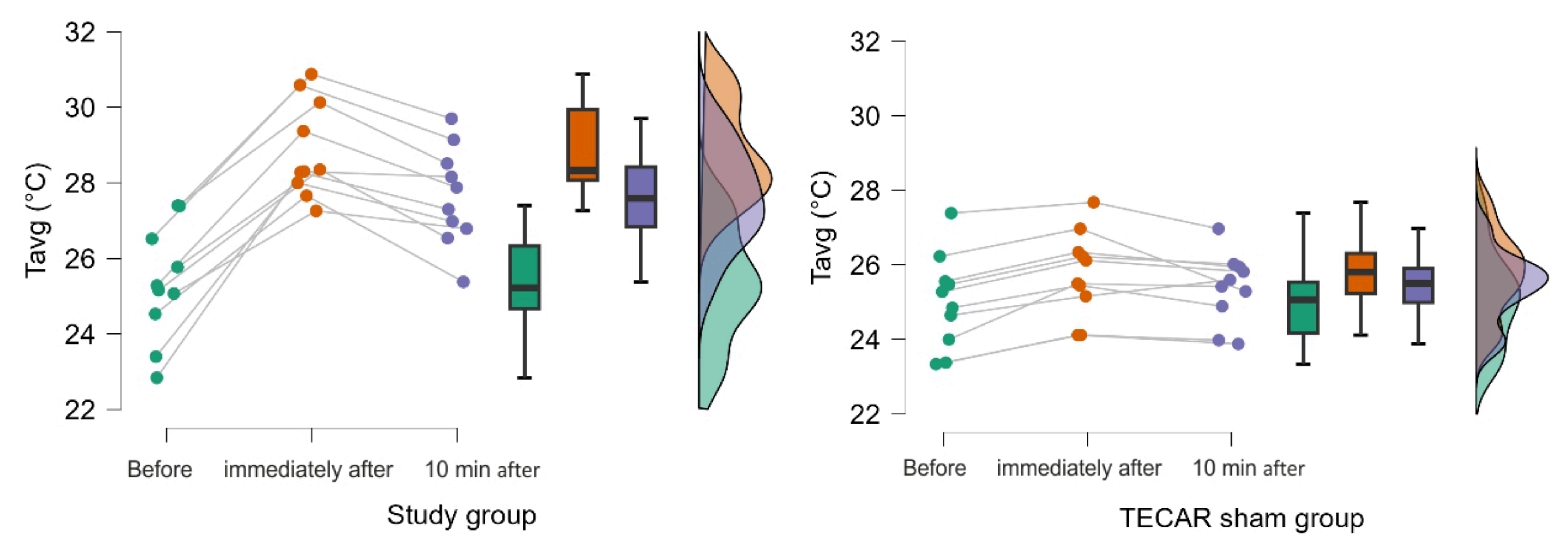
Changes in mean body surface temperature (Tavg) measured before treatment (AVG_pre), immediately after treatment (AVG_post), and 10 min after treatment (AVG_10min) in the study and TECAR sham groups.

Within-group analysis revealed a significant effect of time on body surface temperature in the study group (repeated-measures ANOVA: *p* < 0.001). In contrast, no significant effect of time was observed in the TECAR sham group (repeated-measures ANOVA: *p* = 0.341) (Figure 2).

### 3.2. Palpation Examination

Palpation scores for the longissimus dorsi muscle recorded at the three assessment time points (PAL_pre, PAL_post, and PAL_10min) are presented in Table 2.

In the study group, a significant reduction in muscle tone was observed over time (Friedman’s test: *p* < 0.001). The median palpation score decreased from 3 [2–3] before treatment (PAL_pre) to 0 [0–1] immediately after treatment (PAL_post) and remained unchanged at 0 [0–1] 10 min later (PAL_10min). Post hoc pairwise analysis revealed significant differences between PAL_pre and PAL_post, as well as between PAL_pre and PAL_10min (both *p* = 0.05).

**Figure 3:**
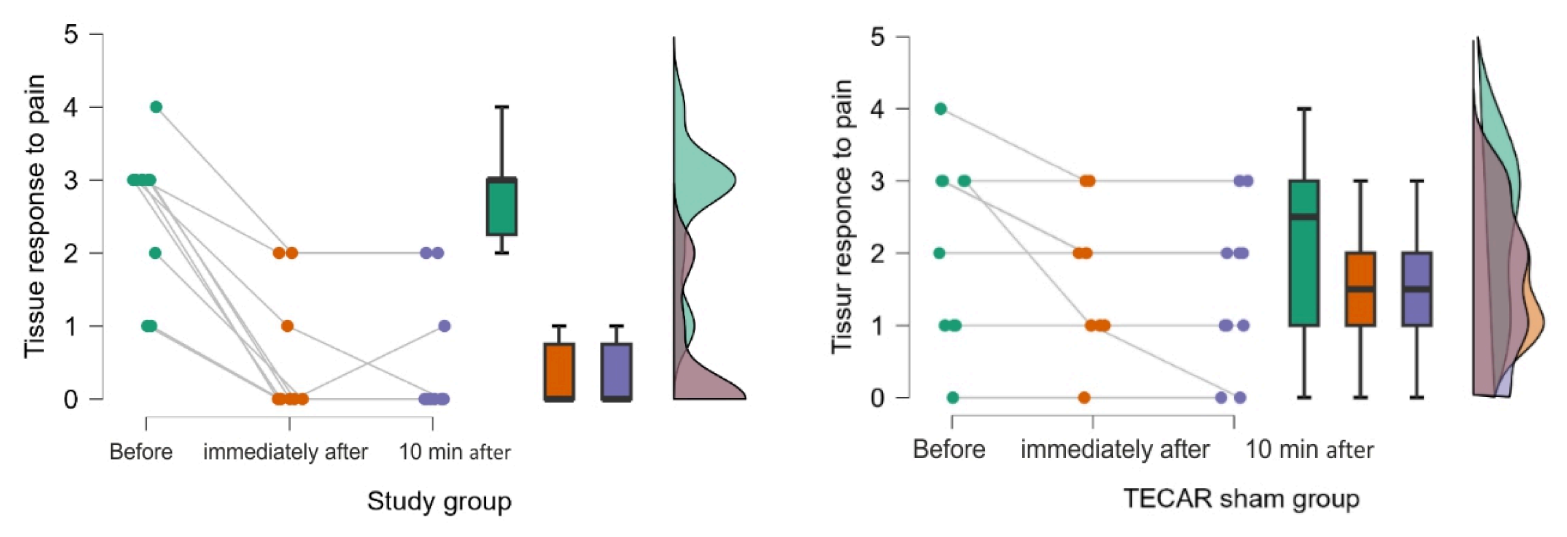
Changes in palpation scores of the longissimus dorsi muscle recorded before treatment (PAL_pre), immediately after treatment (PAL_post), and 10 min after treatment (PAL_10min) in the study and TECAR sham groups.

In the TECAR sham group, a less pronounced reduction in palpation scores was observed (Friedman’s test: *p* = 0.023). The median score decreased from 2.5 [1–3] before treatment to 1.5 [1–2] immediately after treatment and remained at the same level after 10 min. However, post hoc pairwise comparisons between PAL_pre and PAL_post and between PAL_pre and PAL_10min did not reach statistical significance (both *p* = 0.068) (Figure 3).

## 4. Discussion

The present study demonstrates that a single session of TECAR therapy elicits a rapid and measurable increase in body surface temperature over the treated thoracolumbar region of clinically healthy Thoroughbred racehorses, accompanied by a significant reduction in palpation-assessed muscle tone of the longissimus dorsi muscle. Compared with sham-treated controls, horses receiving TECAR therapy exhibited significantly greater increases in body surface temperature. In parallel, palpation-assessed muscle tone of the longissimus dorsi muscle was significantly reduced immediately following treatment. Notably, the thermal response remained detectable 10 min after the intervention, while the reduction in muscle tone was maintained throughout the post-treatment observation period. These findings demonstrate that TECAR therapy induces a measurable thermal response accompanied by a reduction in palpation-assessed muscle tone. The concurrent increase in body surface temperature and decrease in muscle tone observed in the present study is of particular clinical interest, as both responses are consistent with the proposed therapeutic mechanisms of radiofrequency-based physiotherapeutic interventions. The results therefore support the study hypothesis and contribute to the growing body of evidence regarding the thermal response and changes in palpation-assessed muscle tone following TECAR therapy in horses. Given the importance of thoracolumbar muscle function for spinal mobility, locomotor efficiency, and athletic performance in Thoroughbred racehorses, where the thoracolumbar region is subjected to additional loading by the jockey during racing, these findings may have practical implications for the rehabilitation and management of equine athletes.

The increase in body surface temperature observed in the present study is consistent with the fundamental mechanism of TECAR therapy, which relies on the transfer of radiofrequency energy into biological tissues, resulting in endogenous heat generation and subsequent vasodilatory responses. Previous experimental studies have demonstrated that capacitive–resistive electric transfer can increase local blood flow, tissue oxygenation, and regional temperature, thereby enhancing metabolic activity within the treated area [7,30]. The marked increase in body surface temperature observed following TECAR therapy in the present study is therefore consistent with the proposed thermal effects of this modality and provides further evidence of its capacity to induce measurable thermal responses in equine tissues. Importantly, the magnitude of temperature elevation in the TECAR-treated horses substantially exceeded that observed in the sham group, indicating that the thermal response was primarily attributable to active radiofrequency energy transfer rather than to nonspecific factors such as manual applicator movement or environmental conditions. Nevertheless, the slight increase in body surface temperature recorded in the sham group suggests that mechanical stimulation, local friction, or minor environmental influences may have contributed to a limited degree of superficial warming. A comparable trend was observed for palpation scores, although post hoc analyses did not demonstrate statistically significant differences between individual time points in the sham-treated horses. The persistence of elevated body surface temperature 10 min after treatment indicates that the thermal response was maintained beyond the period of active energy application. Whether this reflects sustained physiological mechanisms, such as increased tissue perfusion, or residual physical heat dissipation cannot be determined from the present study. Such prolonged thermal responses may be particularly relevant in equine rehabilitation and sports medicine, where optimization of tissue recovery and muscular function represents an important therapeutic objective.

Notably, comparable thermographic responses have been reported in veterinary studies conducted in dogs, in which TECAR therapy induced a significant increase in superficial tissue temperature that returned to near-baseline values within approximately 60 s after treatment [31]. In contrast, the thermal response observed in the present study persisted throughout the 10 min post-treatment observation period, suggesting a more prolonged thermal effect. This difference may be attributable to species-specific factors, including variations in body size, tissue composition, vascular architecture, and the distribution of radiofrequency energy within the treated tissues. Similar findings have also been reported in horses, where moderate- and high-intensity CRET protocols applied to the thoracolumbar region resulted in significant increases in body surface temperature [22]. Collectively, these observations support the notion that TECAR therapy is capable of eliciting reproducible thermal responses across species, while highlighting potential differences in the duration and magnitude of these effects.

In parallel with the observed thermal response, TECAR therapy was associated with a marked reduction in palpation-assessed muscle tone of the longissimus dorsi muscle. The decrease in palpation scores from moderate muscle tension before treatment to minimal or absent tension immediately afterward suggests that TECAR therapy may exert a clinically relevant influence on the mechanical properties of equine skeletal muscle. This finding is particularly noteworthy because previous equine studies have primarily focused on thermographic or locomotor outcomes, whereas direct assessment of muscle tone following TECAR therapy has received limited attention. The present results are consistent with findings from human rehabilitation research, where TECAR-induced tissue heating has been associated with reductions in muscle stiffness, increased tissue extensibility, improved flexibility, and enhanced recovery of neuromuscular function [5,18,32]. Several mechanisms have been proposed to explain these effects, including increased collagen extensibility, enhanced local circulation, and physiological responses associated with radiofrequency stimulation. Improved tissue perfusion and oxygen delivery may additionally facilitate the clearance of metabolic by-products, thereby promoting muscle relaxation and reducing localized tension. The simultaneous occurrence of increased body surface temperature and reduced palpation-assessed muscle tone observed in the present study supports the hypothesis that TECAR-induced thermal responses may contribute to alterations in muscle mechanical properties. Although a direct causal relationship cannot be established based on the present data, these findings provide preliminary evidence linking TECAR-induced heating with reduced muscle tone in horses and help address a notable gap in the current veterinary literature.

The findings of the present study are in agreement with a growing body of evidence from both human and veterinary medicine demonstrating measurable thermal responses and clinically assessed changes in muscle tone following TECAR therapy. Clinical and experimental investigations have reported beneficial effects of TECAR treatment on pain modulation, musculoskeletal function, tissue recovery, and post-exercise rehabilitation, although direct comparisons between studies remain challenging due to substantial variability in treatment protocols and methodological heterogeneity [1,2,6,33]. Similarly, equine studies evaluating locomotor biomechanics and accelerometric parameters have documented improvements in back flexibility, stride characteristics, and gait-related variables following CRET application [19,20]. While previous investigations have primarily focused on clinical, biomechanical, or performance-related outcomes, the physiological mechanisms underlying these improvements have remained incompletely understood. In the present study, TECAR therapy induced a pronounced increase in body surface temperature accompanied by a significant reduction in palpation-assessed muscle tone, providing objective evidence of acute tissue-level responses to treatment. These findings suggest that TECAR-induced alterations in tissue perfusion and muscle mechanical properties may represent one of the physiological pathways through which improvements in mobility, comfort, and locomotor performance are achieved. Consequently, the present results contribute to a more comprehensive understanding of the thermal responses and changes in palpation-assessed muscle tone following TECAR therapy in horses, thereby expanding the current evidence supporting its application in equine physiotherapy.

Several limitations should be considered when interpreting the findings of the present study. First, the relatively small sample size may limit the generalizability of the results and warrants cautious extrapolation to broader equine populations. Nevertheless, as a pilot investigation, the study provides important preliminary data regarding the acute effects of TECAR therapy in racehorses and may serve as a foundation for future research. Second, muscle tone was assessed using a subjective palpation scoring system rather than objective biomechanical or instrumental methods. Although all examinations were performed by the same blinded veterinarian using a standardized protocol, the possibility of observer-related variability cannot be completely excluded. Future studies incorporating objective techniques, such as myotonometry, elastography, or electromyography, may provide a more comprehensive characterization of TECAR-induced changes in muscle mechanical properties. Another important limitation is that only the immediate effects of a single TECAR treatment session were evaluated. Consequently, the duration of the observed responses and the potential cumulative effects of repeated treatments remain unknown. Previous investigations have suggested that repeated CRET applications may result in more pronounced and sustained clinical benefits over time [21]. Longitudinal studies are therefore needed to determine the duration of the observed thermal responses and changes in palpation-assessed muscle tone, as well as their potential clinical relevance in equine rehabilitation and athletic training. Finally, considerable heterogeneity exists among TECAR treatment protocols reported in the literature. Variations in treatment intensity, duration, application frequency, electrode positioning, and the relative contribution of capacitive and resistive modes may substantially influence therapeutic responses. Experimental evidence indicates that treatment intensity is a key determinant of the magnitude of thermal and physiological effects, with higher intensities producing more pronounced tissue heating [22]. Further well-controlled studies are therefore required to establish evidence-based treatment parameters and to optimize the clinical application of TECAR therapy in equine physiotherapy. Such efforts will be essential for improving treatment standardization and for clarifying the long-term therapeutic value of this modality in equine athletes.

## 5. Conclusions

The present pilot study demonstrated that a single session of TECAR therapy resulted in a rapid increase in body surface temperature over the treated thoracolumbar region of clinically healthy Thoroughbred racehorses, accompanied by a significant reduction in palpation-assessed muscle tone of the longissimus dorsi muscle. These findings support the potential application of TECAR therapy as a non-invasive physiotherapeutic modality in equine sports medicine and rehabilitation.

By demonstrating an association between TECAR-induced thermal responses and changes in palpation-assessed muscle tone, this study contributes novel information to the limited body of evidence regarding the effects of TECAR therapy in horses. Further research involving larger study populations, objective assessments of muscle mechanical properties, and long-term follow-up is warranted to establish optimal treatment protocols and to determine the clinical relevance of these findings in horses undergoing rehabilitation or athletic training.

## Figures and Tables

**Table 1:** Mean body surface temperature (Tavg, °C) measured before treatment (AVG_pre), immediately after treatment (AVG_post), and 10 min after treatment (AVG_10min) in the study and TECAR sham groups.

Time Point	Study Group (*n* = 10) Mean (SD)	TECAR Sham Group (*n* = 10) Mean (SD)	Test Results
AVG_pre (°C)	25.3 (1.5) ^a^ [24.3–26.4]	25.0 (1.3) ^a^ [24.1–25.9]	*p* = 0.61 *g* = 0.19
AVG_post (°C)	28.9 (1.3) ^b^ [28.0–29.8]	25.8 (1.1) ^a^ [25.0–26.6]	*p* <0.001 *g* = 2.47
AVG_10min (°C)	27.6 (1.3) ^b^ [26.7–28.6]	25.4 (0.9) ^a^ [24.7–26.1]	*p* = 0.002 *g* = 1.88
Test results	*p* <0.001 $\eta_{p}^{2}$ = 0.60	*p* = 0.341 *η^2^_p_* = 0.09	**-**

Note: Values are presented as mean (SD) and [95% CI]. p—*p*-value, g—Hedges’ g effect size, $\eta_{p}^{2}$—partial eta-squared. Different superscript letters (^*a*^,^*b*^) within a column indicate statistically significant differences between time points (post hoc test, p<0.05).

**Table 2:** Palpation scores (median [IQR]) of the longissimus dorsi muscle measured before treatment (PAL_pre), immediately after treatment (PAL_post), and 10 min after treatment (PAL_10min).

Group	PAL-pre	PAL_post	PAL_10min	Test Results
All horses (*n* = 20)	3 [1–3]	1 [0–2]	1 [0–2]	*p* < 0.001, *W* = 0.65
Study group	3 [2–3]	0 [0–1]	0 [0–1]	*p* < 0.001, *W* = 0.94
TECAR sham group	2.5 [1–3]	1.5 [1–2]	1.5 [1–2]	*p* = 0.023, *W* = 0.38

Note: Values are presented as median [IQR] (interquartile range). PAL_pre—palpation score before treatment, PAL_post—palpation score immediately after treatment, PAL_10min—palpation score 10 min after treatment. p—*p*-value from the Friedman’s test, W**—**Kendall’s W effect size.

## Data Availability

The original contributions presented in this study are included in the article. Further inquiries can be directed to the corresponding author.
